# Supplementary material for: The Evolution and Characterization of the RNA Interference Pathways in Lophotrochozoa
Source: Genome Biol Evol. 2024 May 7;16(5):evae098. doi: 10.1093/gbe/evae098 (PMC11114477; doi:10.1093/gbe/evae098)
Supplement: evae098_Supplementary_Data [file evae098_supplementary_data.zip › Supplementary_materials.zip!/selected_miRNAs/NoGe_selected_miRNAs/mirdeep_pdf_files/NMRB01002238.1_Notospermus_geniculatus_strain_Ushimado_scaffold2238,_whole_genome_shotgun_sequence_36957.pdf]

Provisional ID	:	NMRB01002238.1_Notospermus_geniculatus_strain_Ushimado_scaffold2238,_whole_genome_shotgun_sequence_36957
Score total	:	846
Score for star read(s)	:	3.9
Score for read counts	:	840.8
Score for mfe	:	1.9
Score for randfold	:	0
Score for cons. seed	:	-0.6
Total read count	:	1661
Mature read count	:	1649
Loop read count	:	0
Star read count	:	12

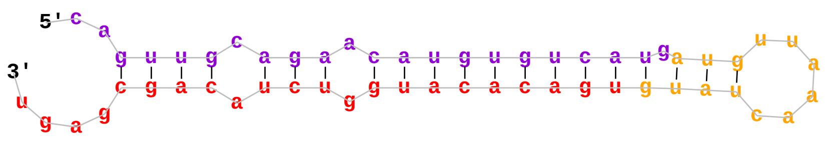

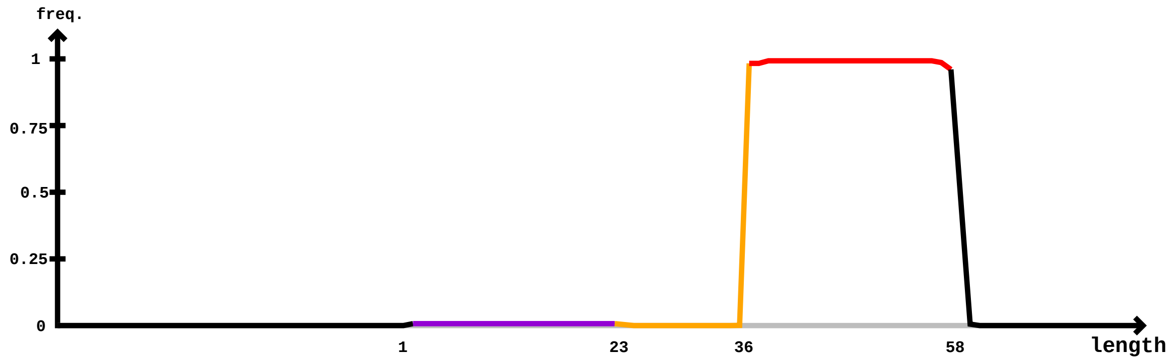


```
                                                   Star                                     Mature
5'- uugaugcaugcgguauagauacugucacgacgcaccaguugcagaacaugugucaugauguuaaacuaugugacacauggucuacagcgaguguguuggcaggguuucaa  -3'    obs
    uugaugcaugcgguauagauacugucacgacgcaccaguugcagaacaugugucaugauguuaaacuaugugacacauggucuacagcgaguguguuggcaggguuucaa        exp
    ...((((.....))))((((.(((((.((((((((..((((.(((.((((((((((.(((.....)))))))))))))).))).)))).))))))))))))).))))...   reads   mm   sample
    .....................................................................Cugacacauggucuacagcgagu..................   1       1    Fm3
    ......................................................................ugacacauggucuacagcga....................   1       0    Fm3
    ......................................................................ugacacauggucuacagcgU....................   2       1    Fm3
    ......................................................................ugacacauggucuacagGga....................   1       1    Fm3
    ......................................................................ugacacauggucuacaCcga....................   1       1    Fm3
    ......................................................................ugacacauggucuacagcgag...................   17      0    Fm3
    ......................................................................ugacacauggucuacagAgag...................   2       1    Fm3
    ......................................................................ugacacauggucuacagUgagu..................   5       1    Fm3
    ......................................................................uCacacauggucuacagcgagu..................   1       1    Fm3
    ......................................................................ugacacauggucuacagGgagu..................   2       1    Fm3
    ......................................................................ugacacauggucuacagAgagu..................   8       1    Fm3
    ......................................................................ugacacauggucuacagcgagA..................   1       1    Fm3
    ......................................................................ugacacauggucuacagcgagu..................   758     0    Fm3
    ......................................................................ugacacauggucuacagcUagu..................   1       1    Fm3
    ......................................................................ugacacauggCcuacagcgagu..................   1       1    Fm3
    ......................................................................ugacacauggucuacaUcgagu..................   1       1    Fm3
    ......................................................................ugacacauggucuacaAcgagu..................   3       1    Fm3
    ......................................................................ugacacauggucuacagcgaguU.................   32      1    Fm3

    ...................................caguugcagaacaugugucaug.....................................................   1       0    Mm3
    ...................................caguugcagaacaugugucaugU....................................................   1       1    Mm3
    ......................................................................ugacacauggucuacagcga....................   1       0    Mm3
    ......................................................................ugacacauggucuacagcgG....................   1       1    Mm3
    ......................................................................ugacacauggucuacagcgag...................   4       0    Mm3
    ......................................................................ugacacauggucuacCgcgagu..................   1       1    Mm3
    ......................................................................ugacacauggucuacagAgagu..................   1       1    Mm3
    ......................................................................ugacacauggucuacagUgagu..................   2       1    Mm3
    ......................................................................ugacacauggucuacagcgagu..................   106     0    Mm3
    ......................................................................ugacacauggucuacaAcgagu..................   1       1    Mm3
    ......................................................................ugacacauggucuacagGgagu..................   1       1    Mm3
    ......................................................................ugacacauggucuacagcgaguU.................   6       1    Mm3
    ......................................................................ugacacauggucuacagcgaguA.................   1       1    Mm3
    ........................................................................acacauggucuacagcgagu..................   1       0    Mm3
```

```
                                   Star                              Mature
uugaugcaugcgguauagauacugucacgacgcaccaguugcagaacaugugucaugauguuaaacuaugugacacauggucuacagcgaguguguugggcaggguuucaa

......................................................................ugacacauggucuacagcga.....................  4    0  Fm1
......................................................................ugacacauggucuacagcgag....................  2    0  Fm1
......................................................................ugacacauggucuacagUgagu...................  1    1  Fm1
......................................................................ugacacauggucuacagcgagu...................  159  0  Fm1
......................................................................ugacacauggucuacagcgagG...................  1    1  Fm1
......................................................................ugacacauggucuacagGgagu...................  1    1  Fm1
......................................................................ugacacauggucuacagcgGgu...................  1    1  Fm1
......................................................................ugacacauggucuacagcgaguU..................  2    1  Fm1

...................................caguugcagaacaugugucaug......................................................  5    0  Fm2
...................................caguugcagaacauguCucaug......................................................  1    1  Fm2
......................................................................ugacacauUgucuacagcgagu...................  1    1  Fm2
......................................................................ugacacauggucuacagGgagu...................  1    1  Fm2
......................................................................ugacacauggucuacagcgagu...................  99   0  Fm2
......................................................................ugacacauggucuacagcgagC...................  1    1  Fm2
......................................................................ugacacauggucuacagUgagu...................  1    1  Fm2

...................................caguugcagaacaugugucaug......................................................  4    0  Mm2
......................................................................ugacacauggucuacagcgag....................  2    0  Mm2
......................................................................ugacacauUgucuacagcgagu...................  1    1  Mm2
......................................................................ugacacauggucuacaAcgagu...................  1    1  Mm2
......................................................................ugacacauggucuacagcgagu...................  292  0  Mm2
......................................................................ugaAacauggucuacagcgagu...................  1    1  Mm2
......................................................................ugacacauggucuacagGgagu...................  1    1  Mm2
......................................................................ugacaUauggucuacagcgagu...................  1    1  Mm2
......................................................................ugacacauggucuacagAgagu...................  3    1  Mm2
......................................................................ugacacauggucuacagcgaguU..................  10   1  Mm2
......................................................................ugacacauggucuacagcgaguA..................  1    1  Mm2
......................................................................ugacacauggucuacagcgaguAu.................  2    1  Mm2
......................................................................ugacacauggucuacagcgaguUu.................  6    1  Mm2
........................................................................acacauggucuacagcgaguUu.................  1    1  Mm2

......................................................................ugacacauggucuacagcgag....................  16   0  Mm1
......................................................................ugacacauggucuacaAcgagu...................  1    1  Mm1
......................................................................ugacacauggucuacagcgagu...................  50   0  Mm1
......................................................................ugacacauggucuacagAgagu...................  1    1  Mm1
......................................................................ugacacauggucuacagcgaguU..................  7    1  Mm1
........................................................................acacauggucuacagcgagu...................  13   0  Mm1
........................................................................acacauggucuacagcgGgu...................  1    1  Mm1
```
